# Supplementary material for: Defining explicit definitions of potentially inappropriate prescriptions for antidiabetic drugs in patients with type 2 diabetes: A systematic review
Source: PLoS One. 2022 Sep 12;17(9):e0274256. doi: 10.1371/journal.pone.0274256 (PMC9467327; doi:10.1371/journal.pone.0274256)
Supplement: S1 Table — (PDF) [file pone.0274256.s001.pdf]

DATABASE	EQUATION
**MEDLINE VIA PUBMED**	(“Diabetes Mellitus”[MeSH Terms] OR "diabet*" [Tiab]) AND ("hypoglycemic agents"[MeSH Terms] OR "vildagliptin"[MeSH Terms] OR "gliclazide"[MeSH Terms] OR "glipizide"[MeSH Terms] OR "pioglitazone"[MeSH Terms] OR "rosiglitazone"[MeSH Terms] OR "liraglutide"[MeSH Terms] OR "canagliflozin"[MeSH Terms] OR "Metformin"[MeSH Terms] OR "Acarbose"[MeSH Terms] OR "Biguanides"[MeSH Terms] OR "Carbutamide"[MeSH Terms] OR "Chlorpropamide"[MeSH Terms] OR "Exenatide"[MeSH Terms] OR "Glyburide"[MeSH Terms] OR "sitagliptin phosphate"[MeSH Terms] OR "Tolazamide"[MeSH Terms] OR "Troglitazone"[MeSH Terms] OR "hypoglycemic agent*"[Title/Abstract] OR "hypoglycaemic agent*"[Title/Abstract] OR "vildagliptin"[Title/Abstract] OR "gliclazide"[Title/Abstract] OR "glipizide"[Title/Abstract] OR "pioglitazone"[Title/Abstract] OR "rosiglitazone"[Title/Abstract] OR "liraglutide"[Title/Abstract] OR "canagliflozin"[Title/Abstract] OR "Metformin"[Title/Abstract] OR "Acarbose"[Title/Abstract] OR "Biguanide*"[Title/Abstract] OR "Carbutamide"[Title/Abstract] OR "Chlorpropamide"[Title/Abstract] OR "Exenatide"[Title/Abstract] OR "Glyburide"[Title/Abstract] OR "sitagliptin phosphate"[Title/Abstract] OR "Tolazamide"[Title/Abstract] OR "Troglitazone"[Title/Abstract] OR "Sitagliptin"[Title/Abstract] OR "sulfonylure*"[Title/Abstract] OR "biguanide derivate"[Title/Abstract] OR "dipeptidyl peptidase iv inhibitor*"[Title/Abstract] OR "alpha glucosidase inhibitor*"[Title/Abstract] OR "repaglinide"[Title/Abstract] OR "semaglutide"[Title/Abstract] OR "dpp 4 inhibitor*"[Title/Abstract] OR "glp 1 agonist*"[Title/Abstract] OR "saxagliptin"[Title/Abstract] OR "sglt 2 inhibitor*"[Title/Abstract] OR "sodium glucose cotransporter 2 inhibitor*"[Title/Abstract] OR "glibenclamide"[Title/Abstract] OR "meglitinide"[Title/Abstract] OR "dulaglutide"[Title/Abstract] OR "alpha glucosidase inhibitor*"[Title/Abstract] OR "dapagliflozin"[Title/Abstract] OR "empagliflozin"[Title/Abstract] OR "ertugliflozin"[Title/Abstract] OR "ipragliflozin"[Title/Abstract] OR "Glimepiride"[Title/Abstract] OR "Glitazone*"[Title/Abstract] OR "glucagon like peptide 1 receptor agonist*"[Title/Abstract] OR "glibornuride"[Title/Abstract] OR "miglitol"[Title/Abstract] OR "antidiabetic*"[Title/Abstract] OR "Tolbutamide" [Title/Abstract] OR "vildagliptin" [Title/Abstract] OR "lixisenatide" [Title/Abstract] OR "Albiglutide" [Title/Abstract] or "alogliptin" [Title/Abstract] OR "sotagliflozin" [Title/Abstract] OR "thiazolidinedion*" [Title/Abstract]) AND ("Potentially Inappropriate Medication List"[MeSH Terms] OR "Inappropriate Prescribing"[MeSH Terms] OR "Appropriate*"[Title/Abstract] OR "Inappropriate*" [Title/Abstract] OR "prescription drug overuse"[MeSH Terms] OR "improper medic*" [TIAB] OR "Potentially Harmful Medic*" [TIAB] OR "prescription omission*" [TIAB] OR "suboptimal prescri*" [TIAB] OR "overprescri*" [TIAB] OR "Over-prescri*" [TIAB] OR "underprescri*" [TIAB] OR "under-prescri*" [TIAB] OR "misprescri*" [TIAB] OR "mis-prescri*" [TIAB] OR "prescribing error*" [TIAB] OR "incorrect prescri*" [TIAB] OR "prescription error*" [TIAB] OR "drug contraindication*" [TIAB] OR "optimal prescri*" [TIAB] OR "correct prescri*" [TIAB])
**Web Of Science**	(TI=("Diabetes Mellitus") OR AB=("Diabetes Mellitus") OR AK=("Diabetes Mellitus") OR TI=("diabet*") OR AB=("diabet*")) AND (TI=("Hypoglyc$emic Agent*") OR AB=("Hypoglyc$emic Agent*") OR AK=("Hypoglyc$emic Agent*") OR TI=("vildagliptin") OR AB=("vildagliptin") OR AK=("vildagliptin") OR TI=("gliclazide") OR AB=("gliclazide") OR AK=("gliclazide") OR TI=("glipizide") OR AB=("glipizide") OR AK=("glipizide") OR TI=("pioglitazone") OR AB=("pioglitazone") OR AK=("pioglitazone") OR TI=("rosiglitazone") OR AB=("rosiglitazone") OR AK=("rosiglitazone") OR TI=("liraglutide") OR AB=("liraglutide") OR AK=("liraglutide") OR TI=("canagliflozin") OR AB=("canagliflozin") OR AK=("canagliflozin") OR TI=("Metformin") OR AB=("Metformin") OR AK=("Metformin") OR TI=("Acarbose") OR AB=("Acarbose") OR AK=("Acarbose") OR TI=("Biguanide*") OR AB=("Biguanide*") OR AK=("Biguanide*") OR TI=("Carbutamide") OR AB=("Carbutamide") OR AK=("Carbutamide") OR TI=("Chlorpropamide") OR AB=("Chlorpropamide") OR AK=("Chlorpropamide") OR TI=("Exenatide") OR AB=("Exenatide") OR AK=("Exenatide") OR TI=("Glyburide") OR AB=("Glyburide") OR AK=("Glyburide") OR TI=("sitagliptin phosphate") OR AB=("sitagliptin phosphate") OR AK=("sitagliptin phosphate") OR TI=("Tolazamide") OR AB=("Tolazamide") OR AK=("Tolazamide") OR TI=("Troglitazone") OR AB=("Troglitazone") OR AK=("Troglitazone") OR TI=("Sitagliptin") OR AB=("Sitagliptin") OR TI=("sulfonylurea*") OR AB=("sulfonylure*") OR TI=("biguanide derivate") OR AB=("biguanide derivate") OR TI=("dipeptidyl peptidase iv inhibitor*") OR AB=("dipeptidyl peptidase iv inhibitor*") OR TI=("alpha glucosidase inhibitor*") OR AB=("alpha glucosidase inhibitor*") OR TI=("repaglinide") OR AB=("repaglinide") OR TI=("semaglutide") OR AB=("semaglutide") OR TI=("dpp 4 inhibitor*") OR AB=("dpp 4 inhibitor*") OR TI=("glp 1 agonist*") OR AB=("glp 1 agonist*") OR TI=("saxagliptin") OR AB=("saxagliptin") OR TI=("sglt 2 inhibitor*") OR AB=("sglt 2 inhibitor*") OR TI=("sodium glucose cotransporter 2 inhibitor*") OR AB=("sodium glucose cotransporter 2 inhibitor*") OR TI=("glibenclamide") OR AB=("glibenclamide") OR TI=("meglitinide") OR AB=("meglitinide") OR TI=("dulaglutide") OR AB=("dulaglutide") OR TI=("alpha glucosidase inhibitor*") OR AB=("alpha glucosidase inhibitor*") OR TI=("dapagliflozin") OR AB=("dapagliflozin") OR TI=("empagliflozin") OR AB=("empagliflozin") OR TI=("ertugliflozin") OR AB=("ertugliflozin") OR TI=("ipragliflozin") OR AB=("ipragliflozin") OR TI=("Glimepiride") OR AB=("Glimepiride") OR TI=("Glitazone*") OR AB=("Glitazone*") OR TI=("glucagon like peptide 1 receptor agonist") OR AB=("glucagon like peptide 1 receptor agonist") OR TI=("glibornuride") OR AB=("glibornuride") OR TI=("miglitol") OR AB=("miglitol") OR TI=("antidiabetic*") OR AB=("antidiabetic*") OR TI=("Tolbutamide") OR AB=("Tolbutamide") OR TI=("vildagliptin") OR AB=("vildagliptin") OR TI=("lixisenatide") OR AB=("lixisenatide") OR TI=("Albiglutide") OR AB=("Albiglutide") OR TI=("alogliptin") OR AB=("alogliptin") OR TI=("sotagliflozin") OR AB=("sotagliflozin") OR TI=("thiazolidinedion*") OR AB=("thiazolidinedion*")) AND (TI=("Potentially Inappropriate Medication List") OR AB=("Potentially Inappropriate Medication List") OR AK=("Potentially Inappropriate Medication List") OR TI=("Inappropriate Prescribing") OR AB=("Inappropriate Prescribing") OR AK=("Inappropriate Prescribing") OR TI=("Appropriate*") OR AB=("Appropriate*") OR TI=("Inappropriate*") OR AB=("Inappropriate*") OR TI=("prescription drug overuse") OR AB=("prescription drug overuse") OR AK=("prescription drug overuse") OR TI=("improper medic*") OR AB=("improper medic*") OR TI=("Potentially Harmful Medic*") OR AB=("Potentially Harmful Medic*") OR TI=("prescription omission*") OR AB=("prescription omission*") OR TI=("suboptimal prescri*") OR AB=("suboptimal prescri*") OR TI=("overprescri*") OR AB=("overprescri*") OR TI=("Over-prescri*") OR AB=("Over-prescri*") OR TI=("underprescri*") OR AB=("underprescri*") OR TI=("under-prescri*") OR AB=("under-prescri*") OR TI=("misprescri*") OR AB=("misprescri*") OR TI=("mis-prescri*") OR AB=("mis-prescri*") OR TI=("prescribing error*") OR AB=("prescribing error*") OR TI=("incorrect prescri*") OR AB=("incorrect prescri*") OR TI=("prescription error*") OR AB=("prescription error*") OR TI=("drug contraindication*") OR AB=("drug contraindication*") OR TI=("optimal prescri*") OR AB=("optimal prescri*") OR TI=("correct prescri*") OR AB=("correct prescri*"))
**SCOPUS**	(TITLE-ABS-KEY ({Diabetes AND Mellitus}) OR TITLE-ABS ("diabet*")) AND (TITLE-ABS-KEY ("hypoglyc?emic AND agent*") OR TITLE-ABS-KEY ({vildagliptin}) OR TITLE-ABS-KEY ({gliclazide}) OR TITLE-ABS-KEY ({glipizide}) OR TITLE-ABS-KEY ({pioglitazone}) OR TITLE-ABS-KEY ({rosiglitazone}) OR TITLE-ABS-KEY ({liraglutide}) OR TITLE-ABS-KEY ({canagliflozin}) OR TITLE-ABS-KEY ({Metformin}) OR TITLE-ABS-KEY ({Acarbose}) OR TITLE-ABS-KEY ("Biguanide*") OR TITLE-ABS-KEY ({Carbutamide}) OR TITLE-ABS-KEY ({Chlorpropamide}) OR TITLE-ABS-KEY ({Exenatide}) OR TITLE-ABS-KEY ({Glyburide}) OR TITLE-ABS-KEY ({sitagliptin AND phosphate}) OR TITLE-ABS-KEY ({Tolazamide}) OR TITLE-ABS-KEY ({Troglitazone}) OR TITLE-ABS ({Sitagliptin}) OR TITLE-ABS ("sulfonylure*") OR TITLE-ABS ({biguanide AND derivate}) OR TITLE-ABS ("dipeptidyl AND peptidase AND iv AND inhibitor*") OR TITLE-ABS ("alpha AND glucosidase AND inhibitor*") OR TITLE-ABS ({repaglinide}) OR TITLE-ABS ({semaglutide}) OR TITLE-ABS ("dpp AND 4 AND inhibitor*") OR TITLE-ABS ("glp AND 1 AND agonist*") OR TITLE-ABS ({saxagliptin}) OR TITLE-ABS ("sglt AND 2 AND inhibitor*") OR TITLE-ABS ("sodium AND glucose AND cotransporter AND 2 AND inhibitor*") OR TITLE-ABS ({glibenclamide}) OR TITLE-ABS ({meglitinide}) OR TITLE-ABS ({dulaglutide}) OR TITLE-ABS ("alpha AND glucosidase AND inhibitor*") OR TITLE-ABS ({dapagliflozin}) OR TITLE-ABS ({empagliflozin}) OR TITLE-ABS ({ertugliflozin}) OR TITLE-ABS ({ipragliflozin}) OR TITLE-ABS ({Glimepiride}) OR TITLE-ABS ("Glitazone*") OR TITLE-ABS ("glucagon AND like AND peptide AND 1 AND receptor AND agonist*") OR TITLE-ABS ({glibornuride}) OR TITLE-ABS ({miglitol}) OR TITLE-ABS ("antidiabetic*") OR TITLE-ABS ({Tolbutamide}) OR TITLE-ABS ({vildagliptin}) OR TITLE-ABS ({lixisenatide}) OR TITLE-ABS ({Albiglutide}) OR TITLE-ABS ({alogliptin}) OR TITLE-ABS ({sotagliflozin}) OR TITLE-ABS ("thiazolidinedion*")) AND (TITLE-ABS-KEY ({Potentially AND Inappropriate AND Medication AND List}) OR TITLE-ABS-KEY ({Inappropriate AND Prescribing}) OR TITLE-ABS ("Appropriate*") OR TITLE-ABS ("Inappropriate*") OR TITLE-ABS ({prescription AND drug AND overuse}) OR TITLE-ABS ("improper AND medic*") OR TITLE-ABS ("Potentially AND Harmful AND Medic*") OR TITLE-ABS ("prescription AND omission*") OR TITLE-ABS ("suboptimal AND prescri*") OR TITLE-ABS ("overprescri*") OR TITLE-ABS ("Over-prescri*") OR TITLE-ABS ("underprescri*") OR TITLE-ABS ("under-prescri*") OR TITLE-ABS ("misprescri*") OR TITLE-ABS ("mis-prescri*") OR TITLE-ABS ("prescribing AND error*") OR TITLE-ABS ("incorrect AND prescri*") OR TITLE-ABS ("prescription AND error*") OR TITLE-ABS ("drug AND contraindication*") OR TITLE-ABS ("optimal AND prescri*") OR TITLE-ABS ("correct AND prescri*"))
**EMBASE**	('Diabetes Mellitus'/exp OR 'diabet*':ti,ab) AND ('vildagliptin'/exp OR 'gliclazide'/exp OR 'glipizide'/exp OR 'pioglitazone'/exp OR 'rosiglitazone'/exp OR 'liraglutide'/exp OR 'canagliflozin'/exp OR 'metformin'/exp OR 'acarbose'/exp OR 'biguanide derivative'/exp OR 'carbutamide'/exp OR 'chlorpropamide'/exp OR 'exendin 4'/exp OR 'glibenclamide'/exp OR 'sitagliptin'/exp OR 'tolazamide'/exp OR 'troglitazone'/exp OR 'hypoglycemic agent*':ti,ab OR 'vildagliptin':ti,ab OR 'gliclazide':ti,ab OR 'glipizide':ti,ab OR 'pioglitazone':ti,ab OR 'rosiglitazone':ti,ab OR 'liraglutide':ti,ab OR 'canagliflozin':ti,ab OR 'metformin':ti,ab OR 'acarbose':ti,ab OR 'biguanide*':ti,ab OR 'carbutamide':ti,ab OR 'chlorpropamide':ti,ab OR 'exenatide':ti,ab OR 'glyburide':ti,ab OR 'sitagliptin phosphate':ti,ab OR 'tolazamide':ti,ab OR 'troglitazone':ti,ab OR 'sitagliptin':ti,ab OR 'sulfonylurea':ti,ab OR 'biguanide derivate':ti,ab OR 'dipeptidyl peptidase iv inhibitor*':ti,ab OR 'repaglinide':ti,ab OR 'semaglutide':ti,ab OR 'dpp 4 inhibitor*':ti,ab OR 'glp 1 agonist*':ti,ab OR 'saxagliptin':ti,ab OR 'sglt 2 inhibitor*':ti,ab OR 'sodium glucose cotransporter 2 inhibitor*':ti,ab OR 'glibenclamide':ti,ab OR 'meglitinide':ti,ab OR 'dulaglutide':ti,ab OR 'alpha glucosidase inhibitor*':ti,ab OR 'dapagliflozin':ti,ab OR 'empagliflozin':ti,ab OR 'ertugliflozin':ti,ab OR 'ipragliflozin':ti,ab OR 'glimepiride':ti,ab OR 'glitazone*':ti,ab OR 'glucagon like peptide 1 receptor agonist*':ti,ab OR 'glibornuride':ti,ab OR 'miglitol':ti,ab OR 'antidiabetic*':ti,ab OR 'Tolbutamide':ti,ab OR 'vildagliptin':ti,ab OR 'lixisenatide':ti,ab OR 'Albiglutide':ti,ab OR 'alogliptin':ti,ab OR 'sotagliflozin':ti,ab OR 'thiazolidinedion*':ti,ab) AND ('potentially inappropriate medication'/exp OR 'inappropriate prescribing'/exp OR 'Appropriate*':ti,ab OR 'Inappropriate*':ti,ab OR 'prescription drug overuse'/exp OR 'improper medic*':ti,ab OR 'Potentially Harmful Medic*':ti,ab OR 'prescription omission*':ti,ab OR 'suboptimal prescri*':ti,ab OR 'overprescri*':ti,ab OR 'Over-prescri*':ti,ab OR 'underprescri*':ti,ab OR 'under-prescri*':ti,ab OR 'misprescri*':ti,ab OR 'mis-prescri*':ti,ab OR 'prescribing error*':ti,ab OR 'incorrect prescri*':ti,ab OR 'prescription error*':ti,ab OR 'drug contraindication*':ti,ab OR 'optimal prescri*':ti,ab OR 'correct prescri*':ti,ab)
