# Supplementary material for: Defining explicit definitions of potentially inappropriate prescriptions for antidiabetic drugs in patients with type 2 diabetes: A systematic review
Source: PLoS One. 2022 Sep 12;17(9):e0274256. doi: 10.1371/journal.pone.0274256 (PMC9467327; doi:10.1371/journal.pone.0274256)
Supplement: S2 Table — (PDF) [file pone.0274256.s002.pdf]

**Abbreviations**	ALP: alkaline phosphatase<br>ALT: alanine aminotransferase<br>ASP: aspartate aminotransferase<br>CKD-EPI: chronic kidney disease epidemiology collaboration<br>CrCl: creatininine clearance<br>DPP-4: dipeptidyl peptidase-4<br>eGFR: estimated glomerular filtration rate<br>GGT: gamma-glutamyl transferase<br>GLP-1 RA: glucagon-like peptide-1 receptor agonist<br>ICD-9-CM: international classification of diseases, ninth revision, clinical modification<br>MDRD: modification of diet in renal disease<br>NYHA: New York Heart Association<br>SGLT-2: sodium-glucose transport protein 2

DRUG CLASS	THE MENTION OF AN EXPLICIT DEFINITION	REFERENCES
Alpha glucosidase inhibitors	Acarbose: contraindicated eGFR (estimated glomerular filtration rate) <30 mL/min/1.73m2	Davies M, Chatterjee S, Khunti K (2016) The treatment of type 2 diabetes in the presence of renal impairment: what we should know about newer therapies. Clin Pharmacol 8:61–81. https://doi.org/10.2147/CPAA.S82008
Alpha glucosidase inhibitors	Severe renal impairment (defined as a creatininine clearance (CrCl) less than 30mL/min or eGFR less than 30mL/min/1,73m2) was identified as contraindication to the prescribing of acarbose	Khalil V, Sajan C, Tsai T, Ma D (2018) Antidiabetics' usage in type 2 diabetes mellitus: Are prescribing guidelines adhered to? A single centre study. Diabetes Metab Syndr 12(5):635–641. https://doi.org/10.1016/j.dsx.2018.04.005
Alpha glucosidase inhibitors	Severe renal impairment (defined as a eGFR less than 30mL/min/1,73m2) was identified as contraindication to the prescribing of acarbose	Khalil V, Sajan C, Tsai T, Ma D (2018) Antidiabetics' usage in type 2 diabetes mellitus: Are prescribing guidelines adhered to? A single centre study. Diabetes Metab Syndr 12(5):635–641. https://doi.org/10.1016/j.dsx.2018.04.005
Associations of antidiabetics drugs	Saxagliptin + metformin were contraindicated for serum creatinine greater than 1.4 mg/dL for women	Melzer-Cohen C, Karasik A, Leuschner PJ, Azuri J, Shalev V, Chodick G (2018) Dose adjustment of metformin and dipeptidyl-peptidase IV inhibitors in diabetic patients with renal dysfunction. Curr Med Res Opin 34(10):1849–1854. https://doi.org/10.1080/03007995.2018.1459529
Associations of antidiabetics drugs	Saxagliptin + metformin were contraindicated for serum creatinine greater than 1.5 mg/dL for men	Melzer-Cohen C, Karasik A, Leuschner PJ, Azuri J, Shalev V, Chodick G (2018) Dose adjustment of metformin and dipeptidyl-peptidase IV inhibitors in diabetic patients with renal dysfunction. Curr Med Res Opin 34(10):1849–1854. https://doi.org/10.1080/03007995.2018.1459529
Associations of antidiabetics drugs	Sitagliptin + metformin were contraindicated for serum creatinine greater than 1.4 mg/dL for women	Melzer-Cohen C, Karasik A, Leuschner PJ, Azuri J, Shalev V, Chodick G (2018) Dose adjustment of metformin and dipeptidyl-peptidase IV inhibitors in diabetic patients with renal dysfunction. Curr Med Res Opin 34(10):1849–1854. https://doi.org/10.1080/03007995.2018.1459529
Associations of antidiabetics drugs	Sitagliptin + metformin were contraindicated for serum creatinine greater than 1.5 mg/dL for men	Melzer-Cohen C, Karasik A, Leuschner PJ, Azuri J, Shalev V, Chodick G (2018) Dose adjustment of metformin and dipeptidyl-peptidase IV inhibitors in diabetic patients with renal dysfunction. Curr Med Res Opin 34(10):1849–1854. https://doi.org/10.1080/03007995.2018.1459529
Associations of antidiabetics drugs	Vildagliptin + metformin were contraindicated for eGFR less than 60 ml/min per 1.73 m2 (eGFR level as calculated by modification of diet in renal disease (MDRD) formula)	Melzer-Cohen C, Karasik A, Leuschner PJ, Azuri J, Shalev V, Chodick G (2018) Dose adjustment of metformin and dipeptidyl-peptidase IV inhibitors in diabetic patients with renal dysfunction. Curr Med Res Opin 34(10):1849–1854. https://doi.org/10.1080/03007995.2018.1459529
Associations of antidiabetics drugs	Thiazolidinediones prescription was defined as potentially inappropriate if they were prescribed as a Thiazolidinediones + metformin combination regimen in patient with a history of renal insufficiency (~ inpatient hospitalization or outpatient visit with a diagnosis of renal insufficiency (ICD-9-CM code=585))	Wen Y-W, Tsai Y-W, Huang W-F, Hsiao F-Y, Chen P-F (2011) The potentially inappropriate prescription of new drug: thiazolidinediones for patients with type II diabetes in Taiwan. Pharmacoepidemiol Drug Saf 20(1):20–29. https://doi.org/10.1002/pds.2010
Associations of antidiabetics drugs	Thiazolidinediones prescription was defined as potentially inappropriate if they were prescribed as a Thiazolidinediones + metformin combination regimen in patients with renal insufficiency	Wen Y-W, Tsai Y-W, Huang W-F, Hsiao F-Y, Chen P-F (2011) The potentially inappropriate prescription of new drug: thiazolidinediones for patients with type II diabetes in Taiwan. Pharmacoepidemiol Drug Saf 20(1):20–29. https://doi.org/10.1002/pds.2010
Biguanides	Metformin if eGFR < 30 ml/min/1.73m2. We calculated eGFR using MDRD formula	Al-Musawe L, Torre C, Guerreiro JP, et al (2021) Drug-drug interactions and inappropriate medicines impact on glycemic control and kidney function in older adults with diabetes-attending specialty care institution. Eur J Clin Pharmacol. https://doi.org/10.1007/s00228-021-03107-y
Biguanides	Metformin: Not recommended for use after 80 years of age	Altuntaş Y (2019) Approach Toward Diabetes Treatment in the Elderly. Sisli Etfal Hastan Tip Bul 53(2):96–102. https://doi.org/10.14744/SEMB.2019.00868
Biguanides	Metformin: contraindicated in eGFR <30 ml/min	Altuntaş Y (2019) Approach Toward Diabetes Treatment in the Elderly. Sisli Etfal Hastan Tip Bul 53(2):96–102. https://doi.org/10.14744/SEMB.2019.00868

Biguanides	Metformin should be avoided in individuals with clinical evidence of hepatic impairment	Bailey T (2013) Options for combination therapy in type 2 diabetes: comparison of the ADA/EASD position statement and AACE/ACE algorithm. Am J Med 126(9 Suppl 1):S10-20. https://doi.org/10.1016/j.amjmed.2013.06.009
Biguanides	Metformin should be avoided in individuals with laboratory evidence of hepatic impairment	Bailey T (2013) Options for combination therapy in type 2 diabetes: comparison of the ADA/EASD position statement and AACE/ACE algorithm. Am J Med 126(9 Suppl 1):S10-20. https://doi.org/10.1016/j.amjmed.2013.06.009
Biguanides	Potentially inappropriate medicine in eldery: Metformin in those aged >85 years old	Caughey GE, Roughead EE, Vitry AI, McDermott RA, Shakib S, Gilbert AL (2010) Comorbidity in the elderly with diabetes: Identification of areas of potential treatment conflicts. Diabetes Res Clin Pract 87(3):385–393. https://doi.org/10.1016/j.diabres.2009.10.019
Biguanides	Metformin administration must be stopped if eGFR falls to <30 mL/min/1.73m2	Davies M, Chatterjee S, Khunti K (2016) The treatment of type 2 diabetes in the presence of renal impairment: what we should know about newer therapies. Clin Pharmacol 8:61–81. https://doi.org/10.2147/CPAA.S82008
Biguanides	Metformin: contraindicated eGFR <45 mL/min/1.73m2	Davies M, Chatterjee S, Khunti K (2016) The treatment of type 2 diabetes in the presence of renal impairment: what we should know about newer therapies. Clin Pharmacol 8:61–81. https://doi.org/10.2147/CPAA.S82008
Biguanides	metformin was contraindicated (CrCl <30 mL/min)	Doody HK, Peterson GM, Watson D, Castelino RL (2015) Retrospective evaluation of potentially inappropriate prescribing in hospitalized patients with renal impairment. Curr Med Res Opin 31(3):525–535. https://doi.org/10.1185/03007995.2015.1010036
Biguanides	Metformin: 2g daily max if CrCl 60–90 mL/minute	Doody HK, Peterson GM, Watson D, Castelino RL (2015) Retrospective evaluation of potentially inappropriate prescribing in hospitalized patients with renal impairment. Curr Med Res Opin 31(3):525–535. https://doi.org/10.1185/03007995.2015.1010036
Biguanides	Metformin: 1g daily max if CrCl 30–60 mL/minute	Doody HK, Peterson GM, Watson D, Castelino RL (2015) Retrospective evaluation of potentially inappropriate prescribing in hospitalized patients with renal impairment. Curr Med Res Opin 31(3):525–535. https://doi.org/10.1185/03007995.2015.1010036
Biguanides	Metformin avoided with an eGFR <30 mL/ min/1.73 m	Goldman-Levine JD (2015) Combination therapy when metformin is not an option for type 2 diabetes. Ann Pharmacother 49(6):688–699. https://doi.org/10.1177/1060028015572653
Biguanides	Metformin should be discontinued if the serum creatinine exceeds 150 μmol/l	Hamilton CA (2012) Pharmacological management of type 2 diabetes mellitus in patients with CKD. J Ren Care 38 Suppl 1:59–66. https://doi.org/10.1111/j.1755-6686.2012.00275.x
Biguanides	Metformin should be discontinued if the eGFR falls below 30 ml/min/1.73 m2	Hamilton CA (2012) Pharmacological management of type 2 diabetes mellitus in patients with CKD. J Ren Care 38 Suppl 1:59–66. https://doi.org/10.1111/j.1755-6686.2012.00275.x
Biguanides	Recommend the following maximum daily doses related to creatinine clearance: 1 g (15 mL/min)	Heaf J (2014) Metformin in chronic kidney disease: time for a rethink. Perit Dial Int 34(4):353–357. https://doi.org/10.3747/pdi.2013.00344
Biguanides	Recommend the following maximum daily doses related to creatinine clearance: 2 g (60 mL/min)	Heaf J (2014) Metformin in chronic kidney disease: time for a rethink. Perit Dial Int 34(4):353–357. https://doi.org/10.3747/pdi.2013.00344
Biguanides	Recommend the following maximum daily doses related to creatinine clearance: 3 g (120 mL/min)	Heaf J (2014) Metformin in chronic kidney disease: time for a rethink. Perit Dial Int 34(4):353–357. https://doi.org/10.3747/pdi.2013.00344
Biguanides	Recommend the following maximum daily doses related to creatinine clearance: 500 mg (15 mL/min)	Heaf J (2014) Metformin in chronic kidney disease: time for a rethink. Perit Dial Int 34(4):353–357. https://doi.org/10.3747/pdi.2013.00344
Biguanides	The dosage of metformin was considered inappropriate in: a dosage higher than 2 gram per day for patients with a CrCl between 60 and 90 mL/min calculating using the Cockcroft-Gault equation	Huang DL, Abrass IB, Young BA (2014) Medication safety and chronic kidney disease in older adults prescribed metformin: a cross-sectional analysis. BMC Nephrol 15:86. https://doi.org/10.1186/1471-2369-15-86
Biguanides	Metformin was considered contraindicated in patients with a CrCl less than 30 mL/min calculating using the Cockcroft-Gault equation	Huang DL, Abrass IB, Young BA (2014) Medication safety and chronic kidney disease in older adults prescribed metformin: a cross-sectional analysis. BMC Nephrol 15:86. https://doi.org/10.1186/1471-2369-15-86
Biguanides	Metformin was considered contraindicated in patients with eGFR less than 30 mL/min per 1.73 m2. using chronic kidney disease epidemiology collaboration (CKD-EPI) equation	Huang DL, Abrass IB, Young BA (2014) Medication safety and chronic kidney disease in older adults prescribed metformin: a cross-sectional analysis. BMC Nephrol 15:86. https://doi.org/10.1186/1471-2369-15-86
Biguanides	Metformin is contraindicated with severe dehydration	Khalil V, Sajan C, Tsai T, Ma D (2018) Antidiabetics' usage in type 2 diabetes mellitus: Are prescribing guidelines adhered to? A single centre study. Diabetes Metab Syndr 12(5):635–641. https://doi.org/10.1016/j.dsx.2018.04.005
Biguanides	Metformin is contraindicated with diabetic ketoacidosis	Khalil V, Sajan C, Tsai T, Ma D (2018) Antidiabetics' usage in type 2 diabetes mellitus: Are prescribing guidelines adhered to? A single centre study. Diabetes Metab Syndr 12(5):635–641. https://doi.org/10.1016/j.dsx.2018.04.005
Biguanides	Metformin is contraindicated with gangrene	Khalil V, Sajan C, Tsai T, Ma D (2018) Antidiabetics' usage in type 2 diabetes mellitus: Are prescribing guidelines adhered to? A single centre study. Diabetes Metab Syndr 12(5):635–641. https://doi.org/10.1016/j.dsx.2018.04.005
Biguanides	Metformin is contraindicated with severe cardiac failure. Severe cardiac failure was identified as documented symptoms indicating stage 3 or 4 heart failure according to the New York Heart Association (NYHA) classifications in the patient's medical histories	Khalil V, Sajan C, Tsai T, Ma D (2018) Antidiabetics' usage in type 2 diabetes mellitus: Are prescribing guidelines adhered to? A single centre study. Diabetes Metab Syndr 12(5):635–641. https://doi.org/10.1016/j.dsx.2018.04.005

Biguanides	Metformin is contraindicated with renal impairment	Khalil V, Sajan C, Tsai T, Ma D (2018) Antidiabetics' usage in type 2 diabetes mellitus: Are prescribing guidelines adhered to? A single centre study. Diabetes Metab Syndr 12(5):635–641. https://doi.org/10.1016/j.dsx.2018.04.005
Biguanides	Metformin is contraindicated with recent myocardial infarction	Khalil V, Sajan C, Tsai T, Ma D (2018) Antidiabetics' usage in type 2 diabetes mellitus: Are prescribing guidelines adhered to? A single centre study. Diabetes Metab Syndr 12(5):635–641. https://doi.org/10.1016/j.dsx.2018.04.005
Biguanides	Metformin is contraindicated with lactic acidosis	Khalil V, Sajan C, Tsai T, Ma D (2018) Antidiabetics' usage in type 2 diabetes mellitus: Are prescribing guidelines adhered to? A single centre study. Diabetes Metab Syndr 12(5):635–641. https://doi.org/10.1016/j.dsx.2018.04.005
Biguanides	Metformin is contraindicated with severe hepatic dysfunction. Sever hepatic dysfunction was defined as biochemical evidence of hypoalbuminaemia and abnormal serum levels of at least two of the following: total bilirubin, alanine aminotransferase (ALT), alkaline phosphatase (ALP) or gamma-glutamyl transferase (GGT)	Khalil V, Sajan C, Tsai T, Ma D (2018) Antidiabetics' usage in type 2 diabetes mellitus: Are prescribing guidelines adhered to? A single centre study. Diabetes Metab Syndr 12(5):635–641. https://doi.org/10.1016/j.dsx.2018.04.005
Biguanides	Metformin is contraindicated with respiratory failure	Khalil V, Sajan C, Tsai T, Ma D (2018) Antidiabetics' usage in type 2 diabetes mellitus: Are prescribing guidelines adhered to? A single centre study. Diabetes Metab Syndr 12(5):635–641. https://doi.org/10.1016/j.dsx.2018.04.005
Biguanides	Metformin is contraindicated with pancreatitis	Khalil V, Sajan C, Tsai T, Ma D (2018) Antidiabetics' usage in type 2 diabetes mellitus: Are prescribing guidelines adhered to? A single centre study. Diabetes Metab Syndr 12(5):635–641. https://doi.org/10.1016/j.dsx.2018.04.005
Biguanides	Metformin prescribed at a dose of 2000 mg one daily to a patient with a calculated CrCl greater than 60 mL/min would be considered appropriate, whereas the same prescription in an individual with a CrCl of less than 60 mL/min would be inappropriate	Khanal A, Peterson GM, Castelino RL, Jose MD (2015) Potentially inappropriate prescribing of renally cleared drugs in elderly patients in community and aged care settings. Drugs Aging 32(5):391–400. https://doi.org/10.1007/s40266-015-0261-1
Biguanides	Metformin is contraindicated in patients with renal insufficiency (eGFR < 60ml/min/1,73m2)	Kosmalski M, Drozdowska A, Sliwinska A, Drzewoski J (2012) Inappropriate metformin prescribing in elderly type 2 diabetes mellitus (T2DM) patients. Adv Med Sci 57(1):65–70. https://doi.org/10.2478/v10039-012-0017-7
Biguanides	Metformin is contraindicated in patients with heart failure. Presence of symptoms identify a III or IV class according to the classification of NYHA	Kosmalski M, Drozdowska A, Sliwinska A, Drzewoski J (2012) Inappropriate metformin prescribing in elderly type 2 diabetes mellitus (T2DM) patients. Adv Med Sci 57(1):65–70. https://doi.org/10.2478/v10039-012-0017-7
Biguanides	Metformin is contraindicated in patients with liver dysfunction. Liver dysfunction was defined as a elevation of the liver enzyme activity (ALT, aspartate aminotransferase (ASP) > 3 fold normal rang and the presence of common symptims of liver dysfontiun)	Kosmalski M, Drozdowska A, Sliwinska A, Drzewoski J (2012) Inappropriate metformin prescribing in elderly type 2 diabetes mellitus (T2DM) patients. Adv Med Sci 57(1):65–70. https://doi.org/10.2478/v10039-012-0017-7
Biguanides	Metformin is contraindicated in patients with respiratory failure	Kosmalski M, Drozdowska A, Sliwinska A, Drzewoski J (2012) Inappropriate metformin prescribing in elderly type 2 diabetes mellitus (T2DM) patients. Adv Med Sci 57(1):65–70. https://doi.org/10.2478/v10039-012-0017-7
Biguanides	Metformin is contraindicated in patients with creatinine clearance <60ml/min	Kosmalski M, Drozdowska A, Sliwinska A, Drzewoski J (2012) Inappropriate metformin prescribing in elderly type 2 diabetes mellitus (T2DM) patients. Adv Med Sci 57(1):65–70. https://doi.org/10.2478/v10039-012-0017-7
Biguanides	A glomerular filtration rate (GFR) of ≤30 mL/min should be an absolute contraindication to Metformin	Kosmalski M, Drozdowska A, Sliwinska A, Drzewoski J (2012) Inappropriate metformin prescribing in elderly type 2 diabetes mellitus (T2DM) patients. Adv Med Sci 57(1):65–70. https://doi.org/10.2478/v10039-012-0017-7
Biguanides	twice-daily dose of 1,000 mg of Metformin is inappropriat in patients with CKD stages 3–5	Lalau J-D, Kajbaf F, Bennis Y, Hurtel-Lemaire A-S, Belpaire F, De Broe ME (2018) Metformin Treatment in Patients With Type 2 Diabetes and Chronic Kidney Disease Stages 3A, 3B, or 4. Diabetes Care 41(3):547–553. https://doi.org/10.2337/dc17-2231
Biguanides	UK guidelines recommend avoiding metformin in people with an eGFR of less than 30 ml/min, with dose reduction advised at 45 ml/min	Mathur S, Zammitt NN, Frier BM (2015) Optimal glycaemic control in elderly people with type 2 diabetes: what does the evidence say? Drug Saf 38(1):17–32. https://doi.org/10.1007/s40264-014-0247-7
Biguanides	Maximal dose of metformin was defined as 1 g for eGFR 30-45 ml/min per 1.73 m2	Melzer-Cohen C, Karasik A, Leuschner PJ, Azuri J, Shalev V, Chodick G (2018) Dose adjustment of metformin and dipeptidyl-peptidase IV inhibitors in diabetic patients with renal dysfunction. Curr Med Res Opin 34(10):1849–1854. https://doi.org/10.1080/03007995.2018.1459529
Biguanides	Maximal dose of metformin was defined as 2 g. for patients with eGFR 45-60 ml/min per 1.73 m2	Melzer-Cohen C, Karasik A, Leuschner PJ, Azuri J, Shalev V, Chodick G (2018) Dose adjustment of metformin and dipeptidyl-peptidase IV inhibitors in diabetic patients with renal dysfunction. Curr Med Res Opin 34(10):1849–1854. https://doi.org/10.1080/03007995.2018.1459529
Biguanides	Maximal dose of metformin was contraindicated for eGFR less than 30 ml/min per 1,73m2	Melzer-Cohen C, Karasik A, Leuschner PJ, Azuri J, Shalev V, Chodick G (2018) Dose adjustment of metformin and dipeptidyl-peptidase IV inhibitors in diabetic patients with renal dysfunction. Curr Med Res Opin 34(10):1849–1854. https://doi.org/10.1080/03007995.2018.1459529

Biguanides	Metformin is contraindicated with creatinine clearance <30 mL/min calculated by MDRD formula	Moon J, Kumar SS, Graham GG, et al (2016) Trends in metformin utilisation and dose appropriateness in Australia. Eur J Clin Pharmacol 72(12):1489–1496. https://doi.org/10.1007/s00228-016-2117-y
Biguanides	Metformin is contraindicated with renal failure or renal dysfunction (creatinine clearance <60 mL/min)	Moon J, Kumar SS, Graham GG, et al (2016) Trends in metformin utilisation and dose appropriateness in Australia. Eur J Clin Pharmacol 72(12):1489–1496. https://doi.org/10.1007/s00228-016-2117-y
Biguanides	Metformin should be ceased in patients with eGFR <30 mL/min	Moon J, Kumar SS, Graham GG, et al (2016) Trends in metformin utilisation and dose appropriateness in Australia. Eur J Clin Pharmacol 72(12):1489–1496. https://doi.org/10.1007/s00228-016-2117-y
Biguanides	Metformin is contraindicated with end-stage kidney disease (creatinine clearance <15 mL/min)	Petersons CJ (2018) Second steps in managing type 2 diabetes. Aust Prescr 41(5):141–144. https://doi.org/10.18773/austprescr.2018.043
Biguanides	dehydration are contraindications of metformin	Rhee SY, Kim HJ, Ko S-H, et al (2017) Monotherapy in patients with type 2 diabetes mellitus. Korean J Intern Med 32(6):959–966. https://doi.org/10.3904/kjim.2017.312
Biguanides	heart failure are contraindications of metformin	Rhee SY, Kim HJ, Ko S-H, et al (2017) Monotherapy in patients with type 2 diabetes mellitus. Korean J Intern Med 32(6):959–966. https://doi.org/10.3904/kjim.2017.312
Biguanides	Hepatic failure are contraindications of metformin	Rhee SY, Kim HJ, Ko S-H, et al (2017) Monotherapy in patients with type 2 diabetes mellitus. Korean J Intern Med 32(6):959–966. https://doi.org/10.3904/kjim.2017.312
Biguanides	Clinical situations such as chronic kidney disease (caution in eGFR < 60 mL/min/1.73 m2, contraindication in eGFR < 30 mL/min/1.73 m2)	Rhee SY, Kim HJ, Ko S-H, et al (2017) Monotherapy in patients with type 2 diabetes mellitus. Korean J Intern Med 32(6):959–966. https://doi.org/10.3904/kjim.2017.312
Biguanides	The use of metformin is contraindicated in patients with liver failure	Scheen AJ, Paquot N (2013) Metformin revisited: a critical review of the benefit-risk balance in at-risk patients with type 2 diabetes. Diabetes Metab 39(3):179–190. https://doi.org/10.1016/j.diabet.2013.02.006
Biguanides	Severe chronic respiratory insufficiency is a contraindication to the use of metformin	Scheen AJ, Paquot N (2013) Metformin revisited: a critical review of the benefit-risk balance in at-risk patients with type 2 diabetes. Diabetes Metab 39(3):179–190. https://doi.org/10.1016/j.diabet.2013.02.006
Biguanides	The recommendations advise clinicians to consider discontinuing metformin in people aged 80	Schlender L, Martinez YV, Adeniji C, et al (2017) Efficacy and safety of metformin in the management of type 2 diabetes mellitus in older adults: a systematic review for the development of recommendations to reduce potentially inappropriate prescribing. BMC Geriatr 17(Suppl 1):227. https://doi.org/10.1186/s12877-017-0574-5
Biguanides	The recommendations advise clinicians to consider discontinuing metformin in people with Glomerular Filtration Rate (GFR) ≤60 ml/min.	Schlender L, Martinez YV, Adeniji C, et al (2017) Efficacy and safety of metformin in the management of type 2 diabetes mellitus in older adults: a systematic review for the development of recommendations to reduce potentially inappropriate prescribing. BMC Geriatr 17(Suppl 1):227. https://doi.org/10.1186/s12877-017-0574-5
Biguanides	Metformin is not recommended to be initiated if the eGFR drops below 45 mL/min/1.73 m2	Triantafylidis LK, Hawley CE, Perry LP, Paik JM (2018) The Role of Deprescribing in Older Adults with Chronic Kidney Disease. Drugs Aging 35(11):973–984. https://doi.org/10.1007/s40266-018-0593-8
Biguanides	Metformin is contraindicated when the eGFR is < 30 mL/min/1.73 m2,	Triantafylidis LK, Hawley CE, Perry LP, Paik JM (2018) The Role of Deprescribing in Older Adults with Chronic Kidney Disease. Drugs Aging 35(11):973–984. https://doi.org/10.1007/s40266-018-0593-8
Dipeptidyl peptidase-4 inhibitors	Saxagliptin: contraindicated eGFR <15 mL/min/1.73m2	Davies M, Chatterjee S, Khunti K (2016) The treatment of type 2 diabetes in the presence of renal impairment: what we should know about newer therapies. Clin Pharmacol 8:61–81. https://doi.org/10.2147/CPAA.S82008
Dipeptidyl peptidase-4 inhibitors	Sitagliptin: 50 mg once dialy if CrCl 30–50 mL/minute	Doody HK, Peterson GM, Watson D, Castelino RL (2015) Retrospective evaluation of potentially inappropriate prescribing in hospitalized patients with renal impairment. Curr Med Res Opin 31(3):525–535. https://doi.org/10.1185/03007995.2015.1010036
Dipeptidyl peptidase-4 inhibitors	Sitagliptin: 25 mg once daily if CrCl <30 mL/minute.	Doody HK, Peterson GM, Watson D, Castelino RL (2015) Retrospective evaluation of potentially inappropriate prescribing in hospitalized patients with renal impairment. Curr Med Res Opin 31(3):525–535. https://doi.org/10.1185/03007995.2015.1010036
Dipeptidyl peptidase-4 inhibitors	Vildagliptin: 50 mg once daily if CrCl <60 mL/minute	Doody HK, Peterson GM, Watson D, Castelino RL (2015) Retrospective evaluation of potentially inappropriate prescribing in hospitalized patients with renal impairment. Curr Med Res Opin 31(3):525–535. https://doi.org/10.1185/03007995.2015.1010036
Dipeptidyl peptidase-4 inhibitors	Dipeptidyl peptidase-4 (DPP-4) inhibitors should not be used in patients with a history of pancreatitis	Goldman-Levine JD (2015) Combination therapy when metformin is not an option for type 2 diabetes. Ann Pharmacother 49(6):688–699. https://doi.org/10.1177/1060028015572653
Dipeptidyl peptidase-4 inhibitors	Patients with creatinine clearance between 5 and 49 ml/min, the dose of saxagliptin should be reduced to 2.5 mg daily	Hamilton CA (2012) Pharmacological management of type 2 diabetes mellitus in patients with CKD. J Ren Care 38 Suppl 1:59–66. https://doi.org/10.1111/j.1755-6686.2012.00275.x
Dipeptidyl peptidase-4 inhibitors	Saxagliptin is not recommended for patients with end-stage renal disease requiring haemodialysis	Hamilton CA (2012) Pharmacological management of type 2 diabetes mellitus in patients with CKD. J Ren Care 38 Suppl 1:59–66. https://doi.org/10.1111/j.1755-6686.2012.00275.x

Dipeptidyl peptidase-4 inhibitors	Pancreatitis were listed as prescribing contraindications to DPP-4 inhibitors	Khalil V, Sajan C, Tsai T, Ma D (2018) Antidiabetics' usage in type 2 diabetes mellitus: Are prescribing guidelines adhered to? A single centre study. Diabetes Metab Syndr 12(5):635–641. https://doi.org/10.1016/j.dsx.2018.04.005
Dipeptidyl peptidase-4 inhibitors	Sulphonylurea listed as prescribing contraindications to DPP-4 inhibitors	Khalil V, Sajan C, Tsai T, Ma D (2018) Antidiabetics' usage in type 2 diabetes mellitus: Are prescribing guidelines adhered to? A single centre study. Diabetes Metab Syndr 12(5):635–641. https://doi.org/10.1016/j.dsx.2018.04.005
Dipeptidyl peptidase-4 inhibitors	For saxagliptin if the eGFR <50 mL/min/1.73m2, the dose should be reduced from 5 mg to 2.5 mg a day	Min T, Davies GI, Rice S, Chess J, Stephens JW (2018) Treatment choices for the glycaemic management of patients with type 2 diabetes and chronic kidney disease: Analysis of the SAIL patient linked dataset. Diabetes Metab Syndr 12(2):123–127. https://doi.org/10.1016/j.dsx.2017.11.002
Dipeptidyl peptidase-4 inhibitors	For sitagliptin the dose should be reduced to 25 mg if the eGFR is <30 mL/min/1.73m2	Min T, Davies GI, Rice S, Chess J, Stephens JW (2018) Treatment choices for the glycaemic management of patients with type 2 diabetes and chronic kidney disease: Analysis of the SAIL patient linked dataset. Diabetes Metab Syndr 12(2):123–127. https://doi.org/10.1016/j.dsx.2017.11.002
Dipeptidyl peptidase-4 inhibitors	For sitagliptin the dose should be reduced from 100 mg to 50 mg with an eGFR 30–49 mL/min/1.73m2	Min T, Davies GI, Rice S, Chess J, Stephens JW (2018) Treatment choices for the glycaemic management of patients with type 2 diabetes and chronic kidney disease: Analysis of the SAIL patient linked dataset. Diabetes Metab Syndr 12(2):123–127. https://doi.org/10.1016/j.dsx.2017.11.002
Dipeptidyl peptidase-4 inhibitors	Sitagliptin should be reduce to 25 mg if eGFR <30 mL/min	Nathan DM, Buse JB, Kahn SE, et al (2013) Rationale and design of the glycemia reduction approaches in diabetes: a comparative effectiveness study (GRADE). Diabetes Care 36(8):2254–2261. https://doi.org/10.2337/dc13-0356
Dipeptidyl peptidase-4 inhibitors	Sitagliptin should be reduce to 50 mg if eGFR <45 mL/min	Nathan DM, Buse JB, Kahn SE, et al (2013) Rationale and design of the glycemia reduction approaches in diabetes: a comparative effectiveness study (GRADE). Diabetes Care 36(8):2254–2261. https://doi.org/10.2337/dc13-0356
Dipeptidyl peptidase-4 inhibitors	DPP-4 inhibitors should not be prescribed to people with a previous history of pancreatic disease	Petersons CJ (2018) Second steps in managing type 2 diabetes. Aust Prescr 41(5):141–144. https://doi.org/10.18773/austprescr.2018.043
Glinides	Nateglinide is contraindicated in renal insufficiency	Altuntaş Y (2019) Approach Toward Diabetes Treatment in the Elderly. Sisli Etfal Hastan Tip Bul 53(2):96–102. https://doi.org/10.14744/SEMB.2019.00868
Glucagon-like peptide-1 receptor agonists	Glucagon-like peptide-1 receptor agonist (GLP-1 RA): Not recommended for use after 75 years of age	Altuntaş Y (2019) Approach Toward Diabetes Treatment in the Elderly. Sisli Etfal Hastan Tip Bul 53(2):96–102. https://doi.org/10.14744/SEMB.2019.00868
Glucagon-like peptide-1 receptor agonists	GLP-1 RA should not be used in patients with coexisting pancreatitis	Bailey T (2013) Options for combination therapy in type 2 diabetes: comparison of the ADA/EASD position statement and AACE/ACE algorithm. Am J Med 126(9 Suppl 1):S10-20. https://doi.org/10.1016/j.amjmed.2013.06.009
Glucagon-like peptide-1 receptor agonists	Albiglutide: contraindicated eGFR <30 mL/min/1.73m2	Davies M, Chatterjee S, Khunti K (2016) The treatment of type 2 diabetes in the presence of renal impairment: what we should know about newer therapies. Clin Pharmacol 8:61–81. https://doi.org/10.2147/CPAA.S82008
Glucagon-like peptide-1 receptor agonists	Dulaglutide: contraindicated eGFR <30 mL/min/1.73m2	Davies M, Chatterjee S, Khunti K (2016) The treatment of type 2 diabetes in the presence of renal impairment: what we should know about newer therapies. Clin Pharmacol 8:61–81. https://doi.org/10.2147/CPAA.S82008
Glucagon-like peptide-1 receptor agonists	Exenatide twice daily: contraindicated eGFR <30 mL/min/1.73m2	Davies M, Chatterjee S, Khunti K (2016) The treatment of type 2 diabetes in the presence of renal impairment: what we should know about newer therapies. Clin Pharmacol 8:61–81. https://doi.org/10.2147/CPAA.S82008
Glucagon-like peptide-1 receptor agonists	Exenatide once weekly: contraindicated eGFR <45 mL/min/1.73m2	Davies M, Chatterjee S, Khunti K (2016) The treatment of type 2 diabetes in the presence of renal impairment: what we should know about newer therapies. Clin Pharmacol 8:61–81. https://doi.org/10.2147/CPAA.S82008
Glucagon-like peptide-1 receptor agonists	Liraglutide: contraindicated eGFR <30 mL/min/1.73m2	Davies M, Chatterjee S, Khunti K (2016) The treatment of type 2 diabetes in the presence of renal impairment: what we should know about newer therapies. Clin Pharmacol 8:61–81. https://doi.org/10.2147/CPAA.S82008
Glucagon-like peptide-1 receptor agonists	Lixisenatide: contraindicated eGFR <30 mL/min/1.73m2	Davies M, Chatterjee S, Khunti K (2016) The treatment of type 2 diabetes in the presence of renal impairment: what we should know about newer therapies. Clin Pharmacol 8:61–81. https://doi.org/10.2147/CPAA.S82008
Glucagon-like peptide-1 receptor agonists	GLP-1 RA inhibitors should not be used in patients with a history of pancreatitis	Goldman-Levine JD (2015) Combination therapy when metformin is not an option for type 2 diabetes. Ann Pharmacother 49(6):688–699. https://doi.org/10.1177/1060028015572653
Glucagon-like peptide-1 receptor agonists	Liraglutide is contraindicated if creatinine clearance less than 30 ml/min.	Hamilton CA (2012) Pharmacological management of type 2 diabetes mellitus in patients with CKD. J Ren Care 38 Suppl 1:59–66. https://doi.org/10.1111/j.1755-6686.2012.00275.x
Glucagon-like peptide-1 receptor agonists	Severe renal impairment (defined as a creatininine clearance (CrCl) less than 30mL/min) was identified as contraindication to the prescribing of glucagon like peptide 1 agonists	Khalil V, Sajan C, Tsai T, Ma D (2018) Antidiabetics' usage in type 2 diabetes mellitus: Are prescribing guidelines adhered to? A single centre study. Diabetes Metab Syndr 12(5):635–641. https://doi.org/10.1016/j.dsx.2018.04.005

Glucagon-like peptide-1 receptor agonists	Severe renal impairment (defined as a eGFR less than 30mL/min/1,73m2) was identified as contraindication to the prescribing of glucagon like peptide 1 agonists	Khalil V, Sajan C, Tsai T, Ma D (2018) Antidiabetics' usage in type 2 diabetes mellitus: Are prescribing guidelines adhered to? A single centre study. Diabetes Metab Syndr 12(5):635–641. https://doi.org/10.1016/j.dsx.2018.04.005
Glucagon-like peptide-1 receptor agonists	Exenatide use is not recommended in patients with severe renal impairment (creatinine clearance [CrCl] <30 mL/min)	McCormack PL (2014) Exenatide twice daily: a review of its use in the management of patients with type 2 diabetes mellitus. Drugs 74(3):325–351. https://doi.org/10.1007/s40265-013-0172-6
Glucagon-like peptide-1 receptor agonists	Exenatide use is not recommended in patients with end-stage renal disease	McCormack PL (2014) Exenatide twice daily: a review of its use in the management of patients with type 2 diabetes mellitus. Drugs 74(3):325–351. https://doi.org/10.1007/s40265-013-0172-6
Glucagon-like peptide-1 receptor agonists	Exenatide: CrCl <30 mL/ min not recommended	Meece J (2017) The Role of the Pharmacist in Managing Type 2 Diabetes with Glucagon-Like Peptide-1 Receptor Agonists as Add-On Therapy. Adv Ther 34(3):638–657. https://doi.org/10.1007/s12325-017-0491-1
Oral antidiabetic agents	Oral diabetic agents, except metformin: deprescription aged ≥80 years (frail)	Rodríguez-Pérez A, Alfaro-Lara ER, Albiñana-Perez S, et al (2017) Novel tool for deprescribing in chronic patients with multimorbidity: List of Evidence-Based Deprescribing for Chronic Patients criteria: LESS-CHRON criteria. Geriatr Gerontol Int 17(11):2200–2207. https://doi.org/10.1111/ggi.13062
Sodium-glucose transport protein 2 inhibitors	Sodium-glucose transport protein 2 (SGLT-2) inhibitors: Not recommended for use after 75 years of age	Altuntaş Y (2019) Approach Toward Diabetes Treatment in the Elderly. Sisli Etfal Hastan Tip Bul 53(2):96–102. https://doi.org/10.14744/SEMB.2019.00868
Sodium-glucose transport protein 2 inhibitors	Canagliflozin should not be initiated (and should be discontinued) in patients with an eGFR<45 mL/min/1.73 m2	Blonde L, Dipp S, Cadena D (2018) Combination Glucose-Lowering Therapy Plans in T2DM: Case-Based Considerations. Adv Ther 35(7):939–965. https://doi.org/10.1007/s12325-018-0694-0
Sodium-glucose transport protein 2 inhibitors	The dose of canagliflozin should be limited to 100 mg once daily in patients with an eGFR of 45 to < 60 mL/min/1.73 m2.	Blonde L, Dipp S, Cadena D (2018) Combination Glucose-Lowering Therapy Plans in T2DM: Case-Based Considerations. Adv Ther 35(7):939–965. https://doi.org/10.1007/s12325-018-0694-0
Sodium-glucose transport protein 2 inhibitors	Treatment with dapagliflozin should not be initiated (and should be discontinued) in patients with an eGFR<60 mL/min/1.73 m2	Blonde L, Dipp S, Cadena D (2018) Combination Glucose-Lowering Therapy Plans in T2DM: Case-Based Considerations. Adv Ther 35(7):939–965. https://doi.org/10.1007/s12325-018-0694-0
Sodium-glucose transport protein 2 inhibitors	Empagliflozin should not be initiated (and should be discontinued) in patients with an eGFR<45 mL/min/1.73 m2	Blonde L, Dipp S, Cadena D (2018) Combination Glucose-Lowering Therapy Plans in T2DM: Case-Based Considerations. Adv Ther 35(7):939–965. https://doi.org/10.1007/s12325-018-0694-0
Sodium-glucose transport protein 2 inhibitors	Ertugliflozin should not be initiated in patients with an eGFR of 30 to <60 mL/min/ 1.73 m2, or used in patients with an eGFR<30 mL/min/1.73 m2. In patients with an eGFR of 30 to <60 mL/min/1.73 m2, ongoing use of ertugliflozin is not recommended.	Blonde L, Dipp S, Cadena D (2018) Combination Glucose-Lowering Therapy Plans in T2DM: Case-Based Considerations. Adv Ther 35(7):939–965. https://doi.org/10.1007/s12325-018-0694-0
Sodium-glucose transport protein 2 inhibitors	Canagliflozin: contraindicated eGFR <45 mL/min/1.73m2	Davies M, Chatterjee S, Khunti K (2016) The treatment of type 2 diabetes in the presence of renal impairment: what we should know about newer therapies. Clin Pharmacol 8:61–81. https://doi.org/10.2147/CPAA.S82008
Sodium-glucose transport protein 2 inhibitors	Dapagliflozin: contraindicated eGFR <60 mL/min/1.73m2	Davies M, Chatterjee S, Khunti K (2016) The treatment of type 2 diabetes in the presence of renal impairment: what we should know about newer therapies. Clin Pharmacol 8:61–81. https://doi.org/10.2147/CPAA.S82008
Sodium-glucose transport protein 2 inhibitors	Empagliflozin: contraindicated eGFR <45 mL/min/1.73m2	Davies M, Chatterjee S, Khunti K (2016) The treatment of type 2 diabetes in the presence of renal impairment: what we should know about newer therapies. Clin Pharmacol 8:61–81. https://doi.org/10.2147/CPAA.S82008
Sodium-glucose transport protein 2 inhibitors	Canagliflozin is limited to 100 mg once daily for those with an eGFR of 45 to <60 mL/ min/1.73 m2.	Goldman-Levine JD (2015) Combination therapy when metformin is not an option for type 2 diabetes. Ann Pharmacother 49(6):688–699. https://doi.org/10.1177/1060028015572653
Sodium-glucose transport protein 2 inhibitors	Canagliflozin should not be used in patients with eGFR <45 mL/min/1.73 m2	Goldman-Levine JD (2015) Combination therapy when metformin is not an option for type 2 diabetes. Ann Pharmacother 49(6):688–699. https://doi.org/10.1177/1060028015572653
Sodium-glucose transport protein 2 inhibitors	Dapagliflozin should not be used in patients with eGFR <60 mL/min/1.73 m2	Goldman-Levine JD (2015) Combination therapy when metformin is not an option for type 2 diabetes. Ann Pharmacother 49(6):688–699. https://doi.org/10.1177/1060028015572653
Sodium-glucose transport protein 2 inhibitors	Empagliflozin should not be used in patients with eGFR <45 mL/min/1.73 m2	Goldman-Levine JD (2015) Combination therapy when metformin is not an option for type 2 diabetes. Ann Pharmacother 49(6):688–699. https://doi.org/10.1177/1060028015572653
Sodium-glucose transport protein 2 inhibitors	Sodium glucose transport inhibitors is contraindicated with renal impairment	Khalil V, Sajan C, Tsai T, Ma D (2018) Antidiabetics' usage in type 2 diabetes mellitus: Are prescribing guidelines adhered to? A single centre study. Diabetes Metab Syndr 12(5):635–641. https://doi.org/10.1016/j.dsx.2018.04.005
Sulfonylureas	Chlorpropamide with type 2 diabetes mellitus	Al Aqqad SMH, Chen LL, Shafie AA, Hassali MA, Tangiisuran B (2014) The use of potentially inappropriate medications and changes in quality of life among older nursing home residents. Clin Interv Aging 9:201–207. https://doi.org/10.2147/CIA.S52356
Sulfonylureas	Glibenclamide with type 2 diabetes mellitus	Al Aqqad SMH, Chen LL, Shafie AA, Hassali MA, Tangiisuran B (2014) The use of potentially inappropriate medications and changes in quality of life among older nursing home residents. Clin Interv Aging 9:201–207. https://doi.org/10.2147/CIA.S52356

Sulfonylureas	Glyburide in type 2 diabetes mellitus	Al Khaja KAJ, Ahmed Isa H, Veeramuthu S, Sequeira RP (2018) Potentially Inappropriate Prescribing in Older Adults with Hypertension or Diabetes Mellitus and Hypertension in a Primary Care Setting in Bahrain. Med Princ Pract 27(3):241–249. https://doi.org/10.1159/000488055
Sulfonylureas	Sulphonylureas with a long duration of action with type 2 diabetes mellitus	Al-Musawe L, Torre C, Guerreiro JP, et al (2021) Drug-drug interactions and inappropriate medicines impact on glycemic control and kidney function in older adults with diabetes-attending specialty care institution. Eur J Clin Pharmacol. https://doi.org/10.1007/s00228-021-03107-y
Sulfonylureas	Potentially inappropriate medicine in eldery: Glibenclamide in elderly (>65 years old)	Caughey GE, Roughead EE, Vitry AI, McDermott RA, Shakib S, Gilbert AL (2010) Comorbidity in the elderly with diabetes: Identification of areas of potential treatment conflicts. Diabetes Res Clin Pract 87(3):385–393. https://doi.org/10.1016/j.diabres.2009.10.019
Sulfonylureas	Potentially inappropriate medicine in eldery: Glimepiride in elderly (>65 years old)	Caughey GE, Roughead EE, Vitry AI, McDermott RA, Shakib S, Gilbert AL (2010) Comorbidity in the elderly with diabetes: Identification of areas of potential treatment conflicts. Diabetes Res Clin Pract 87(3):385–393. https://doi.org/10.1016/j.diabres.2009.10.019
Sulfonylureas	Chlorpropamide with type 2 diabetes mellitus	Chandrasekhar D, Samjas M, pattani D (2019) Evaluation of potentially inappropriate medications among hospitalized geriatric patients in tertiary care referral hospital using STOPP/START criteria. Clinical Epidemiology and Global Health 7(3):268–273. https://doi.org/10.1016/j.cegh.2018.10.008
Sulfonylureas	Glibenclamide with type 2 diabetes mellitus	Chandrasekhar D, Samjas M, pattani D (2019) Evaluation of potentially inappropriate medications among hospitalized geriatric patients in tertiary care referral hospital using STOPP/START criteria. Clinical Epidemiology and Global Health 7(3):268–273. https://doi.org/10.1016/j.cegh.2018.10.008
Sulfonylureas	Chlorpropamide with type 2 diabetes mellitus	Chen LL, Tangiisuran B, Shafie AA, Hassali MAA (2012) Evaluation of potentially inappropriate medications among older residents of Malaysian nursing homes. Int J Clin Pharm 34(4):596–603. https://doi.org/10.1007/s11096-012-9651-1
Sulfonylureas	Glibenclamide with type 2 diabetes mellitus	Chen LL, Tangiisuran B, Shafie AA, Hassali MAA (2012) Evaluation of potentially inappropriate medications among older residents of Malaysian nursing homes. Int J Clin Pharm 34(4):596–603. https://doi.org/10.1007/s11096-012-9651-1
Sulfonylureas	Chlorpropamide with type 2 diabetes mellitus	Conejos Miquel MD, Sánchez Cuervo M, Delgado Silveira E, et al (2010) Potentially inappropriate drug prescription in older subjects across health care settings. European Geriatric Medicine 1(1):9–14. https://doi.org/10.1016/j.eurger.2009.12.002
Sulfonylureas	Glibenclamide with type 2 diabetes mellitus	Conejos Miquel MD, Sánchez Cuervo M, Delgado Silveira E, et al (2010) Potentially inappropriate drug prescription in older subjects across health care settings. European Geriatric Medicine 1(1):9–14. https://doi.org/10.1016/j.eurger.2009.12.002
Sulfonylureas	Glibenclamide: contraindicated eGFR <30 mL/min/1.73m2	Davies M, Chatterjee S, Khunti K (2016) The treatment of type 2 diabetes in the presence of renal impairment: what we should know about newer therapies. Clin Pharmacol 8:61–81. https://doi.org/10.2147/CPAA.S82008
Sulfonylureas	Gliclazide: contraindicated eGFR <30 mL/min/1.73m2	Davies M, Chatterjee S, Khunti K (2016) The treatment of type 2 diabetes in the presence of renal impairment: what we should know about newer therapies. Clin Pharmacol 8:61–81. https://doi.org/10.2147/CPAA.S82008
Sulfonylureas	Glimepiride: contraindicated eGFR <30 mL/min/1.73m2	Davies M, Chatterjee S, Khunti K (2016) The treatment of type 2 diabetes in the presence of renal impairment: what we should know about newer therapies. Clin Pharmacol 8:61–81. https://doi.org/10.2147/CPAA.S82008
Sulfonylureas	Glipizide: contraindicated eGFR <30 mL/min/1.73m2	Davies M, Chatterjee S, Khunti K (2016) The treatment of type 2 diabetes in the presence of renal impairment: what we should know about newer therapies. Clin Pharmacol 8:61–81. https://doi.org/10.2147/CPAA.S82008
Sulfonylureas	Glibenclamide: avoid use in renal impairment	Doody HK, Peterson GM, Watson D, Castelino RL (2015) Retrospective evaluation of potentially inappropriate prescribing in hospitalized patients with renal impairment. Curr Med Res Opin 31(3):525–535. https://doi.org/10.1185/03007995.2015.1010036
Sulfonylureas	Glibenclamide with type 2 diabetes mellitus	Fadare JO, Desalu OO, Obimakinde AM, Adeoti AO, Agboola SM, Aina FO (2015) Prevalence of inappropriate medication prescription in the elderly in Nigeria: A comparison of Beers and STOPP criteria. JRS 27(4):177–189. https://doi.org/10.3233/JRS-150660
Sulfonylureas	Chlorpropamide with type 2 diabetes mellitus (risk of prolonged hypoglycaemia)	Formiga F, Vidal X, Agustí A, et al (2016) Inappropriate prescribing in elderly people with diabetes admitted to hospital. Diabet Med 33(5):655–662. https://doi.org/10.1111/dme.12894
Sulfonylureas	Chlorpropamide (Diabinese) It has a prolonged half-life in elderly patients and could cause prolonged hypoglycemia.	Formiga F, Vidal X, Agustí A, et al (2016) Inappropriate prescribing in elderly people with diabetes admitted to hospital. Diabet Med 33(5):655–662. https://doi.org/10.1111/dme.12894
Sulfonylureas	Glibenclamide with type 2 diabetes mellitus (risk of prolonged hypoglycaemia)	Formiga F, Vidal X, Agustí A, et al (2016) Inappropriate prescribing in elderly people with diabetes admitted to hospital. Diabet Med 33(5):655–662. https://doi.org/10.1111/dme.12894

Sulfonylureas	Sulfonylureas should be avoided in older individuals > 65 years old	Gagnon M-E, Sirois C, Simard M, Roux B, Plante C (2020) Potentially inappropriate medications in older individuals with diabetes: A population-based study in Quebec, Canada. Primary Care Diabetes 14(5):529–537. https://doi.org/10.1016/j.pcd.2020.03.003
Sulfonylureas	Chlorpropamide with type 2 diabetes mellitus (risk of prolonged hypoglycaemia)	Gallagher PF, O'Connor MN, O'Mahony D (2011) Prevention of Potentially Inappropriate Prescribing for Elderly Patients: A Randomized Controlled Trial Using STOPP/START Criteria. Clin Pharmacol Ther 89(6):845–854. https://doi.org/10.1038/clpt.2011.44
Sulfonylureas	Glibenclamide with type 2 diabetes mellitus	Gallagher PF, O'Connor MN, O'Mahony D (2011) Prevention of Potentially Inappropriate Prescribing for Elderly Patients: A Randomized Controlled Trial Using STOPP/START Criteria. Clin Pharmacol Ther 89(6):845–854. https://doi.org/10.1038/clpt.2011.44
Sulfonylureas	Sulphonylureas: Inappropriate treatment was defined as patients meeting one or more of the following seven characteristics/risk factors: age >= 75	Giorda CB, Orsi E, De Cosmo S, et al (2020) Prescription of Sulphonylureas among Patients with Type 2 Diabetes Mellitus in Italy: Results from the Retrospective, Observational Multicentre Cross-Sectional SUSCIPE (Sulphonyl_UreaS_Correct_Internal_Prescription_Evaluation) Study. Diabetes Ther 11(9):2105–2119. https://doi.org/10.1007/s13300-020-00871-5
Sulfonylureas	Sulphonylureas: Inappropriate treatment was defined as patients meeting one or more of the following seven characteristics/risk factors: cognitive impairment.	Giorda CB, Orsi E, De Cosmo S, et al (2020) Prescription of Sulphonylureas among Patients with Type 2 Diabetes Mellitus in Italy: Results from the Retrospective, Observational Multicentre Cross-Sectional SUSCIPE (Sulphonyl_UreaS_Correct_Internal_Prescription_Evaluation) Study. Diabetes Ther 11(9):2105–2119. https://doi.org/10.1007/s13300-020-00871-5
Sulfonylureas	Sulphonylureas: Inappropriate treatment was defined as patients meeting one or more of the following seven characteristics/risk factors: history of heart failure, unstable angina, coronary heart disease, stroke, or myocardial infarction	Giorda CB, Orsi E, De Cosmo S, et al (2020) Prescription of Sulphonylureas among Patients with Type 2 Diabetes Mellitus in Italy: Results from the Retrospective, Observational Multicentre Cross-Sectional SUSCIPE (Sulphonyl_UreaS_Correct_Internal_Prescription_Evaluation) Study. Diabetes Ther 11(9):2105–2119. https://doi.org/10.1007/s13300-020-00871-5
Sulfonylureas	Sulphonylureas: Inappropriate treatment was defined as patients meeting one or more of the following seven characteristics/risk factors: historyof severe hypoglycemia (conventionally defined as an episode that requires assistance from another person)	Giorda CB, Orsi E, De Cosmo S, et al (2020) Prescription of Sulphonylureas among Patients with Type 2 Diabetes Mellitus in Italy: Results from the Retrospective, Observational Multicentre Cross-Sectional SUSCIPE (Sulphonyl_UreaS_Correct_Internal_Prescription_Evaluation) Study. Diabetes Ther 11(9):2105–2119. https://doi.org/10.1007/s13300-020-00871-5
Sulfonylureas	Sulphonylureas: Inappropriate treatment was defined as patients meeting one or more of the following seven characteristics/risk factors: obesity (set as Body mass index (BMI) > 30 kg/m2, in accordance with the Associazione Medici Diabetologi e della Società Italiana di Diabetologia (AMD–SID) and World Health Organization definitions (WHO))	Giorda CB, Orsi E, De Cosmo S, et al (2020) Prescription of Sulphonylureas among Patients with Type 2 Diabetes Mellitus in Italy: Results from the Retrospective, Observational Multicentre Cross-Sectional SUSCIPE (Sulphonyl_UreaS_Correct_Internal_Prescription_Evaluation) Study. Diabetes Ther 11(9):2105–2119. https://doi.org/10.1007/s13300-020-00871-5
Sulfonylureas	Sulphonylureas: Inappropriate treatment was defined as patients meeting one or more of the following seven characteristics/risk factors: chronic renal insufficiency (moderate to severe or requiring dialysis)	Giorda CB, Orsi E, De Cosmo S, et al (2020) Prescription of Sulphonylureas among Patients with Type 2 Diabetes Mellitus in Italy: Results from the Retrospective, Observational Multicentre Cross-Sectional SUSCIPE (Sulphonyl_UreaS_Correct_Internal_Prescription_Evaluation) Study. Diabetes Ther 11(9):2105–2119. https://doi.org/10.1007/s13300-020-00871-5
Sulfonylureas	Sulphonylureas: Inappropriate treatment was defined as patients meeting one or more of the following seven characteristics/risk factors: cognitive impairment; and risky occupation (bus/taxi/train driver, working at height).	Giorda CB, Orsi E, De Cosmo S, et al (2020) Prescription of Sulphonylureas among Patients with Type 2 Diabetes Mellitus in Italy: Results from the Retrospective, Observational Multicentre Cross-Sectional SUSCIPE (Sulphonyl_UreaS_Correct_Internal_Prescription_Evaluation) Study. Diabetes Ther 11(9):2105–2119. https://doi.org/10.1007/s13300-020-00871-5
Sulfonylureas	For patients with creatinine clearance less than 30 ml/min chlorpropamide should be avoided	Hamilton CA (2012) Pharmacological management of type 2 diabetes mellitus in patients with CKD. J Ren Care 38 Suppl 1:59–66. https://doi.org/10.1111/j.1755-6686.2012.00275.x
Sulfonylureas	For patients with creatinine clearance less than 30 ml/min glibenclimide and should be avoided	Hamilton CA (2012) Pharmacological management of type 2 diabetes mellitus in patients with CKD. J Ren Care 38 Suppl 1:59–66. https://doi.org/10.1111/j.1755-6686.2012.00275.x
Sulfonylureas	Glibenclamide inappropriate - Risk of protracted hypoglycemia in eldery patiens	Harasani K, Xhafaj D, Qipo O (2020) Prevalence and types of potentially inappropriate prescriptions among older and middle-aged community-dwelling Albanian patients. JRS 31(1):5–13. https://doi.org/10.3233/JRS-195052
Sulfonylureas	Oral sulphonylurea with long duration (glibenclamide) in the management of diabetes for Middle-aged People	Harasani K, Xhafaj D, Qipo O (2020) Prevalence and types of potentially inappropriate prescriptions among older and middle-aged community-dwelling Albanian patients. JRS 31(1):5–13. https://doi.org/10.3233/JRS-195052
Sulfonylureas	Long-acting SUs glibenclamide is contraindicated in those whose creatinine clearance is < 40 ml/min, The Cockroft-Gault formula was used in our study	Holstein A, Hammer C, Hahn M, Kulamadayil N-S-A, Kovacs P (2010) Severe sulfonylurea-induced hypoglycemia: a problem of uncritical prescription and deficiencies of diabetes care in geriatric patients. Expert Opin Drug Saf 9(5):675–681. https://doi.org/10.1517/14740338.2010.492777

Sulfonylureas	Long-acting SUs glimepiride is contraindicated in those whose creatinine clearance is < 40 ml/min. The Cockroft-Gault formula was used in our study	Holstein A, Hammer C, Hahn M, Kulamadayil N-S-A, Kovacs P (2010) Severe sulfonylurea-induced hypoglycemia: a problem of uncritical prescription and deficiencies of diabetes care in geriatric patients. Expert Opin Drug Saf 9(5):675–681. https://doi.org/10.1517/14740338.2010.492777
Sulfonylureas	Sulphonylurea is contraindicated with diabetic ketoacidosis	Khalil V, Sajan C, Tsai T, Ma D (2018) Antidiabetics' usage in type 2 diabetes mellitus: Are prescribing guidelines adhered to? A single centre study. Diabetes Metab Syndr 12(5):635–641. https://doi.org/10.1016/j.dsx.2018.04.005
Sulfonylureas	Sulphonylurea is contraindicated with severe hepatic impairment. Sever hepatic dysfunction was defined as biochemical evidence of hypoalbuminaemia and abnormal serum levels of at least two of the following: total bilirubin, ALT, ALP or CGT	Khalil V, Sajan C, Tsai T, Ma D (2018) Antidiabetics' usage in type 2 diabetes mellitus: Are prescribing guidelines adhered to? A single centre study. Diabetes Metab Syndr 12(5):635–641. https://doi.org/10.1016/j.dsx.2018.04.005
Sulfonylureas	Sulphonylurea is contraindicated with severe renal impairment, (defined as a creatininine clearance (CrCl) less than 30mL/min or eGFR less than 30mL/min/1,73m2)	Khalil V, Sajan C, Tsai T, Ma D (2018) Antidiabetics' usage in type 2 diabetes mellitus: Are prescribing guidelines adhered to? A single centre study. Diabetes Metab Syndr 12(5):635–641. https://doi.org/10.1016/j.dsx.2018.04.005
Sulfonylureas	Glibenclamide is considered a potentially inappropriate prescription for the adult population over 65 years of age	Machado-Alba JE, Machado-Duque ME, Gaviria-Mendoza A (2020) Time to modification of antidiabetic therapy in patients over the age of 65 years with newly diagnosed diabetes mellitus. Diabetes Res Clin Pract 162:108090. https://doi.org/10.1016/j.diabres.2020.108090
Sulfonylureas	Glibenclamide should not be used in people over the age of 60 years.	Mathur S, Zammitt NN, Frier BM (2015) Optimal glycaemic control in elderly people with type 2 diabetes: what does the evidence say? Drug Saf 38(1):17–32. https://doi.org/10.1007/s40264-014-0247-7
Sulfonylureas	Chlorpropamide with type 2 diabetes mellitus (risk of prolonged hypoglycemia)	Mori ALPM, Carvalho RC, Aguiar PM, et al (2017) Potentially inappropriate prescribing and associated factors in elderly patients at hospital discharge in Brazil: a cross-sectional study. Int J Clin Pharm 39(2):386–393. https://doi.org/10.1007/s11096-017-0433-7
Sulfonylureas	Glibenclamide with type 2 diabetes mellitus (risk of prolonged hypoglycemia)	Mori ALPM, Carvalho RC, Aguiar PM, et al (2017) Potentially inappropriate prescribing and associated factors in elderly patients at hospital discharge in Brazil: a cross-sectional study. Int J Clin Pharm 39(2):386–393. https://doi.org/10.1007/s11096-017-0433-7
Sulfonylureas	Glibenclamide with type 2 diabetes mellitus (risk of prolonged hypoglycaemia)	Nascimento MMG, Ribeiro AQ, Pereira ML, Soares AC, Loyola Filho AI de, Dias-Junior CAC (2014) Identification of inappropriate prescribing in a Brazilian nursing home using STOPP/START screening tools and the Beers' Criteria. Braz J Pharm Sci 50(4):911–918. https://doi.org/10.1590/S1984-82502014000400027
Sulfonylureas	Medications to Avoid When Possible in Older Adults: Glyburide	Pretorius RW, Gataric G, Swedlund SK, Miller JR (2013) Reducing the risk of adverse drug events in older adults. Am Fam Physician 87(5):331–336
Sulfonylureas	Patients considered at increased risk of hypoglycaemia – were on glyburide with a calculated creatinine clearance of < 50 ml/min	Seidu S, Kunutsor SK, Topsever P, Hambling CE, Cos FX, Khunti K (2019) Deintensification in older patients with type 2 diabetes: A systematic review of approaches, rates and outcomes. Diabetes Obes Metab 21(7):1668–1679. https://doi.org/10.1111/dom.13724
Sulfonylureas	Avoid glyburide and use glipizide in patients with a calculated creatinine clearance (CrCl) of less than 50 mL per minute	Skoff RA, Waterbury NV, Shaw RF, Egge JA, Cantrell M (2011) Glycemic control and hypoglycemia in Veterans Health Administration patients converted from glyburide to glipizide. J Manag Care Pharm 17(9):664–671. https://doi.org/10.18553/jmcp.2011.17.9.664
Sulfonylureas	Glibenclamide for type 2 diabetes mellitus	Zaal RJ, Ebbers S, Borms M, et al (2016) Medication review using a Systematic Tool to Reduce Inappropriate Prescribing (STRIP) in adults with an intellectual disability: A pilot study. Research in Developmental Disabilities 55:132–142. https://doi.org/10.1016/j.ridd.2016.03.014
Thiazolidinediones	Pioglitazones: Not recommended for use after 75 years of age	Altuntaş Y (2019) Approach Toward Diabetes Treatment in the Elderly. Sisli Etfal Hastan Tip Bul 53(2):96–102. https://doi.org/10.14744/SEMB.2019.00868
Thiazolidinediones	Potentially inappropriate medicine in eldery: Thiazolidinediones (glitazones) in chronic heart failure	Caughey GE, Roughead EE, Vitry AI, McDermott RA, Shakib S, Gilbert AL (2010) Comorbidity in the elderly with diabetes: Identification of areas of potential treatment conflicts. Diabetes Res Clin Pract 87(3):385–393. https://doi.org/10.1016/j.diabres.2009.10.019
Thiazolidinediones	Pioglitazone should not be used in patients with heart failure	Hamilton CA (2012) Pharmacological management of type 2 diabetes mellitus in patients with CKD. J Ren Care 38 Suppl 1:59–66. https://doi.org/10.1111/j.1755-6686.2012.00275.x
Thiazolidinediones	Thiazolidinediones is contraindicated with severe heart failure. Severe cardiac failure was identified as documented symptoms indicating stage 3 or 4 heart failure according to the NYHA classifications in the patient's medical histories	Khalil V, Sajan C, Tsai T, Ma D (2018) Antidiabetics' usage in type 2 diabetes mellitus: Are prescribing guidelines adhered to? A single centre study. Diabetes Metab Syndr 12(5):635–641. https://doi.org/10.1016/j.dsx.2018.04.005
Thiazolidinediones	Thiazolidinediones is contraindicated with diabetic ketoacidosis	Khalil V, Sajan C, Tsai T, Ma D (2018) Antidiabetics' usage in type 2 diabetes mellitus: Are prescribing guidelines adhered to? A single centre study. Diabetes Metab Syndr 12(5):635–641. https://doi.org/10.1016/j.dsx.2018.04.005
Thiazolidinediones	Thiazolidinedioness are contraindicated in patients with known heart failure	Mathur S, Zammitt NN, Frier BM (2015) Optimal glycaemic control in elderly people with type 2 diabetes: what does the evidence say? Drug Saf 38(1):17–32. https://doi.org/10.1007/s40264-014-0247-7

Thiazolidinediones	Thiazolidinediones prescription was defined as potentially inappropriate if it was prescribed to patients under 18 years old	Wen Y-W, Tsai Y-W, Huang W-F, Hsiao F-Y, Chen P-F (2011) The potentially inappropriate prescription of new drug: thiazolidinediones for patients with type II diabetes in Taiwan. Pharmacoepidemiol Drug Saf 20(1):20–29. https://doi.org/10.1002/pds.2010
Thiazolidinediones	Thiazolidinediones prescription was defined as potentially inappropriate if it was prescribed to patients with a history of hospitalization for heart failure (~1 hospitalization with main diagnosis of heart failure (ICD-9-CM code=402.01, 402.11, 402.91, 404.01, 404.11, 404.91, or 428)) before Thiazolidinediones was prescribed	Wen Y-W, Tsai Y-W, Huang W-F, Hsiao F-Y, Chen P-F (2011) The potentially inappropriate prescription of new drug: thiazolidinediones for patients with type II diabetes in Taiwan. Pharmacoepidemiol Drug Saf 20(1):20–29. https://doi.org/10.1002/pds.2010
Thiazolidinediones	Thiazolidinediones prescription was defined as potentially inappropriate if it patients with a history of hospitalization for hepatic insufficiency (~three hospitalizations or outpatient visits with main diagnosis of hepatic insufficiency (ICD-9-CM code=571 or 572))	Wen Y-W, Tsai Y-W, Huang W-F, Hsiao F-Y, Chen P-F (2011) The potentially inappropriate prescription of new drug: thiazolidinediones for patients with type II diabetes in Taiwan. Pharmacoepidemiol Drug Saf 20(1):20–29. https://doi.org/10.1002/pds.2010
