# Supplementary material for: Defining explicit definitions of potentially inappropriate prescriptions for antidiabetic drugs in patients with type 2 diabetes: A systematic review
Source: PLoS One. 2022 Sep 12;17(9):e0274256. doi: 10.1371/journal.pone.0274256 (PMC9467327; doi:10.1371/journal.pone.0274256)
Supplement: S3 Table — (PDF) [file pone.0274256.s003.pdf]

Drug class	Aggregated definitions of PIPADs not related to renal function or age	References
Biguanides	Metformin is potentially inappropriate in patient with cardiac dysfunction	Khalil V, Sajan C, Tsai T, Ma D (2018) Antidiabetics' usage in type 2 diabetes mellitus: Are prescribing guidelines adhered to? A single centre study. Diabetes Metab Syndr 12(5):635–641. https://doi.org/10.1016/j.dsx.2018.04.005
Biguanides	Metformin is potentially inappropriate in patient with cardiac dysfunction	Kosmalski M, Drozdowska A, Sliwinska A, Drzewoski J (2012) Inappropriate metformin prescribing in elderly type 2 diabetes mellitus (T2DM) patients. Adv Med Sci 57(1):65–70. https://doi.org/10.2478/v10039-012-0017-7
Biguanides	Metformin is potentially inappropriate in patient with cardiac dysfunction	Rhee SY, Kim HJ, Ko S-H, et al (2017) Monotherapy in patients with type 2 diabetes mellitus. Korean J Intern Med 32(6):959–966. https://doi.org/10.3904/kjim.2017.312
Biguanides	Metformin is potentially inappropriate in patient with liver dysfunction	Kosmalski M, Drozdowska A, Sliwinska A, Drzewoski J (2012) Inappropriate metformin prescribing in elderly type 2 diabetes mellitus (T2DM) patients. Adv Med Sci 57(1):65–70. https://doi.org/10.2478/v10039-012-0017-7
Biguanides	Metformin is potentially inappropriate in patient with liver dysfunction	Khalil V, Sajan C, Tsai T, Ma D (2018) Antidiabetics' usage in type 2 diabetes mellitus: Are prescribing guidelines adhered to? A single centre study. Diabetes Metab Syndr 12(5):635–641. https://doi.org/10.1016/j.dsx.2018.04.005
Biguanides	Metformin is potentially inappropriate in patient with liver dysfunction	Rhee SY, Kim HJ, Ko S-H, et al (2017) Monotherapy in patients with type 2 diabetes mellitus. Korean J Intern Med 32(6):959–966. https://doi.org/10.3904/kjim.2017.312
Biguanides	Metformin is potentially inappropriate in patient with liver dysfunction	Scheen AJ, Paquot N (2013) Metformin revisited: a critical review of the benefit-risk balance in at-risk patients with type 2 diabetes. Diabetes Metab 39(3):179–190. https://doi.org/10.1016/j.diabet.2013.02.006
Biguanides	Metformin is potentially inappropriate in patient with liver dysfunction	Bailey T (2013) Options for combination therapy in type 2 diabetes: comparison of the ADA/EASD position statement and AACE/ACE algorithm. Am J Med 126(9 Suppl 1):S10-20. https://doi.org/10.1016/j.amjmed.2013.06.009
Biguanides	Metformin is potentially inappropriate in patient with respiratory dysfunction	Khalil V, Sajan C, Tsai T, Ma D (2018) Antidiabetics' usage in type 2 diabetes mellitus: Are prescribing guidelines adhered to? A single centre study. Diabetes Metab Syndr 12(5):635–641. https://doi.org/10.1016/j.dsx.2018.04.005
Biguanides	Metformin is potentially inappropriate in patient with respiratory dysfunction	Kosmalski M, Drozdowska A, Sliwinska A, Drzewoski J (2012) Inappropriate metformin prescribing in elderly type 2 diabetes mellitus (T2DM) patients. Adv Med Sci 57(1):65–70. https://doi.org/10.2478/v10039-012-0017-7
Biguanides	Metformin is potentially inappropriate in patient with respiratory dysfunction	Scheen AJ, Paquot N (2013) Metformin revisited: a critical review of the benefit-risk balance in at-risk patients with type 2 diabetes. Diabetes Metab 39(3):179–190. https://doi.org/10.1016/j.diabet.2013.02.006
Biguanides	Metformin is potentially inappropriate in patient with diabetic ketoacidosis	Khalil V, Sajan C, Tsai T, Ma D (2018) Antidiabetics' usage in type 2 diabetes mellitus: Are prescribing guidelines adhered to? A single centre study. Diabetes Metab Syndr 12(5):635–641. https://doi.org/10.1016/j.dsx.2018.04.005
Biguanides	Metformin is potentially inappropriate in patient with gangrene	Khalil V, Sajan C, Tsai T, Ma D (2018) Antidiabetics' usage in type 2 diabetes mellitus: Are prescribing guidelines adhered to? A single centre study. Diabetes Metab Syndr 12(5):635–641. https://doi.org/10.1016/j.dsx.2018.04.005
Biguanides	Metformin is potentially inappropriate in patient with lactic acidosis	Khalil V, Sajan C, Tsai T, Ma D (2018) Antidiabetics' usage in type 2 diabetes mellitus: Are prescribing guidelines adhered to? A single centre study. Diabetes Metab Syndr 12(5):635–641. https://doi.org/10.1016/j.dsx.2018.04.005
Biguanides	Metformin is potentially inappropriate in patient with pancreatitis	Khalil V, Sajan C, Tsai T, Ma D (2018) Antidiabetics' usage in type 2 diabetes mellitus: Are prescribing guidelines adhered to? A single centre study. Diabetes Metab Syndr 12(5):635–641. https://doi.org/10.1016/j.dsx.2018.04.005
Biguanides	Metformin is potentially inappropriate in patient with dehydration	Khalil V, Sajan C, Tsai T, Ma D (2018) Antidiabetics' usage in type 2 diabetes mellitus: Are prescribing guidelines adhered to? A single centre study. Diabetes Metab Syndr 12(5):635–641. https://doi.org/10.1016/j.dsx.2018.04.005
Biguanides	Metformin is potentially inappropriate in patient with dehydration	Rhee SY, Kim HJ, Ko S-H, et al (2017) Monotherapy in patients with type 2 diabetes mellitus. Korean J Intern Med 32(6):959–966. https://doi.org/10.3904/kjim.2017.312

Dipeptidyl peptidase-4 inhibitors	Dipeptidyl peptidase-4 inhibitors is potentially inappropriate in patient with pancreatitits dysfunction	Khalil V, Sajan C, Tsai T, Ma D (2018) Antidiabetics’ usage in type 2 diabetes mellitus: Are prescribing guidelines adhered to? A single centre study. Diabetes Metab Syndr 12(5):635–641. https://doi.org/10.1016/j.dsx.2018.04.005
Dipeptidyl peptidase-4 inhibitors	Dipeptidyl peptidase-4 inhibitors is potentially inappropriate in patient with pancreatitits dysfunction	Goldman-Levine JD (2015) Combination therapy when metformin is not an option for type 2 diabetes. Ann Pharmacother 49(6):688–699. https://doi.org/10.1177/1060028015572653
Dipeptidyl peptidase-4 inhibitors	Dipeptidyl peptidase-4 inhibitors is potentially inappropriate in patient with pancreatitits dysfunction	Petersons CJ (2018) Second steps in managing type 2 diabetes. Aust Prescr 41(5):141–144. https://doi.org/10.18773/austprescr.2018.043
Dipeptidyl peptidase-4 inhibitors	Dipeptidyl peptidase-4 inhibitors is potentially inappropriate in patient with sulfonylurea	Khalil V, Sajan C, Tsai T, Ma D (2018) Antidiabetics’ usage in type 2 diabetes mellitus: Are prescribing guidelines adhered to? A single centre study. Diabetes Metab Syndr 12(5):635–641. https://doi.org/10.1016/j.dsx.2018.04.005
Glucagon-like peptide-1 receptor agonists	Glucagon-like peptide-1 receptor agonists is potentially inappropriate in patient with pancreatitits	Bailey T (2013) Options for combination therapy in type 2 diabetes: comparison of the ADA/EASD position statement and AACE/ACE algorithm. Am J Med 126(9 Suppl 1):S10-20. https://doi.org/10.1016/j.amjmed.2013.06.009
Glucagon-like peptide-1 receptor agonists	Glucagon-like peptide-1 receptor agonists is potentially inappropriate in patient with pancreatitits	Goldman-Levine JD (2015) Combination therapy when metformin is not an option for type 2 diabetes. Ann Pharmacother 49(6):688–699. https://doi.org/10.1177/1060028015572653
Sulfonylureas	Sulfonylureas is potentially inappropriate in patient with history of heart failure, unstable angina, coronary heart disease, stroke, or myocardial infarction	Giorda CB, Orsi E, De Cosmo S, et al (2020) Prescription of Sulphonylureas among Patients with Type 2 Diabetes Mellitus in Italy: Results from the Retrospective, Observational Multicentre Cross-Sectional SUSCIPE (Sulphonyl_UreaS_Correct_Internal_Prescription_Evaluation) Study. Diabetes Ther 11(9):2105–2119. https://doi.org/10.1007/s13300-020-00871-5
Sulfonylureas	Sulphonylurea is potentially inappropriate in patient with severe hepatic impairment defined as biochemical evidence of hypoalbuminaemia is potentially inappropriate in patient with abnormal serum levels of at least two of the following: total bilirubin, alanine aminotransferase (ALT), alkaline phosphatase (ALP) or gamma-glutamyl transferase (GGT)	Khalil V, Sajan C, Tsai T, Ma D (2018) Antidiabetics’ usage in type 2 diabetes mellitus: Are prescribing guidelines adhered to? A single centre study. Diabetes Metab Syndr 12(5):635–641. https://doi.org/10.1016/j.dsx.2018.04.005
Sulfonylureas	Sulfonylureas is potentially inappropriate in patient with patients in patient with cognitive impairment	Giorda CB, Orsi E, De Cosmo S, et al (2020) Prescription of Sulphonylureas among Patients with Type 2 Diabetes Mellitus in Italy: Results from the Retrospective, Observational Multicentre Cross-Sectional SUSCIPE (Sulphonyl_UreaS_Correct_Internal_Prescription_Evaluation) Study. Diabetes Ther 11(9):2105–2119. https://doi.org/10.1007/s13300-020-00871-5
Sulfonylureas	Sulfonylureas is potentially inappropriate in patient with patients in patient with historyof severe hypoglycemia (conventionally defined as an episode that requires assistance from another person)	Giorda CB, Orsi E, De Cosmo S, et al (2020) Prescription of Sulphonylureas among Patients with Type 2 Diabetes Mellitus in Italy: Results from the Retrospective, Observational Multicentre Cross-Sectional SUSCIPE (Sulphonyl_UreaS_Correct_Internal_Prescription_Evaluation) Study. Diabetes Ther 11(9):2105–2119. https://doi.org/10.1007/s13300-020-00871-5
Sulfonylureas	Sulfonylureas is potentially inappropriate in patient with patients in patient with obesity (set as body mass index (BMI) > 30 kg/m2,inaccordance in patient with the AMD–SID is potentially inappropriate in patient with World Health Organization definitions)	Giorda CB, Orsi E, De Cosmo S, et al (2020) Prescription of Sulphonylureas among Patients with Type 2 Diabetes Mellitus in Italy: Results from the Retrospective, Observational Multicentre Cross-Sectional SUSCIPE (Sulphonyl_UreaS_Correct_Internal_Prescription_Evaluation) Study. Diabetes Ther 11(9):2105–2119. https://doi.org/10.1007/s13300-020-00871-5
Sulfonylureas	Sulfonylureas is potentially inappropriate in patient with patients in patient with risky occupation (bus/taxi/train driver, working at height)	Giorda CB, Orsi E, De Cosmo S, et al (2020) Prescription of Sulphonylureas among Patients with Type 2 Diabetes Mellitus in Italy: Results from the Retrospective, Observational Multicentre Cross-Sectional SUSCIPE (Sulphonyl_UreaS_Correct_Internal_Prescription_Evaluation) Study. Diabetes Ther 11(9):2105–2119. https://doi.org/10.1007/s13300-020-00871-5
Sulfonylureas	Sulphonylureas is potentially inappropriate in patient with diabetic ketoacidosis	Khalil V, Sajan C, Tsai T, Ma D (2018) Antidiabetics’ usage in type 2 diabetes mellitus: Are prescribing guidelines adhered to? A single centre study. Diabetes Metab Syndr 12(5):635–641. https://doi.org/10.1016/j.dsx.2018.04.005
Thiazolidinediones	Thiazolidinediones is potentially inappropriate in patient with cardiac dysfunction	Khalil V, Sajan C, Tsai T, Ma D (2018) Antidiabetics’ usage in type 2 diabetes mellitus: Are prescribing guidelines adhered to? A single centre study. Diabetes Metab Syndr 12(5):635–641. https://doi.org/10.1016/j.dsx.2018.04.005
Thiazolidinediones	Thiazolidinediones is potentially inappropriate in patient with cardiac dysfunction	Hamilton CA (2012) Pharmacological management of type 2 diabetes mellitus in patients with CKD. J Ren Care 38 Suppl 1:59–66. https://doi.org/10.1111/j.1755-6686.2012.00275.x
Thiazolidinediones	Thiazolidinediones is potentially inappropriate in patient with cardiac dysfunction	Mathur S, Zammitt NN, Frier BM (2015) Optimal glycaemic control in elderly people with type 2 diabetes: what does the evidence say? Drug Saf 38(1):17–32. https://doi.org/10.1007/s40264-014-0247-7

Thiazolidinediones	Thiazolidinediones is potentially inappropriate in patient with cardiac dysfunction	Wen Y-W, Tsai Y-W, Huang W-F, Hsiao F-Y, Chen P-F (2011) The potentially inappropriate prescription of new drug: thiazolidinediones for patients with type II diabetes in Taiwan. Pharmacoepidemiol Drug Saf 20(1):20–29. https://doi.org/10.1002/pds.2010
Thiazolidinediones	Thiazolidinediones is potentially inappropriate in patient with diabetic ketoacidosis	Khalil V, Sajan C, Tsai T, Ma D (2018) Antidiabetics' usage in type 2 diabetes mellitus: Are prescribing guidelines adhered to? A single centre study. Diabetes Metab Syndr 12(5):635–641. https://doi.org/10.1016/j.dsx.2018.04.005
Thiazolidinediones	Thiazolidinediones is potentially inappropriate in patient with patients in patient with a history of hospitalization for hepatic insufficiency (~three hospitalizations or outpatient visits in patient with main diagnosis of hepatic insufficiency (international classification of diseases, ninth revision, clinical modification (ICD-9-CM) code=571 or 572))	Wen Y-W, Tsai Y-W, Huang W-F, Hsiao F-Y, Chen P-F (2011) The potentially inappropriate prescription of new drug: thiazolidinediones for patients with type II diabetes in Taiwan. Pharmacoepidemiol Drug Saf 20(1):20–29. https://doi.org/10.1002/pds.2010
